# Fruit Consumption Habits and Apple Preferences of University Students in Poland

**DOI:** 10.3390/foods14122073

**Published:** 2025-06-12

**Authors:** Paweł Kraciński, Paulina Stolarczyk, Weronika Czerwińska, Bożena Nosecka

**Affiliations:** 1 Department of Economics and Organization of Enterprises, Faculty of Economics, Institute of Economics and Finance, Warsaw University of Life Sciences SGGW, 02-787 Warsaw, Poland; 2 Department of Development Policy and Marketing, Faculty of Economics, Institute of Economics and Finance, Warsaw University of Life Sciences SGGW, ul. Nowoursynowska 166, 02-787 Warsaw, Poland; paulina_stolarczyk@sggw.edu.pl; 3 Department of Economics of Agricultural and Horticultural Holdings, Institute of Agricultural and Food Economics—National Research Institute, ul. Świętokrzyska 20, 00-002 Warsaw, Poland; weronika.czerwinska@ierigz.waw.pl (W.C.); bozena.nosecka@ierigz.waw.pl (B.N.)

**Keywords:** fruit preferences, apple color preferences, fruit purchasing preferences, pricing of apples

## Abstract

The aim of this study was to investigate the preferences of young adults in the Polish apple market in response to the declining consumption of these fruits. To address the research questions, a study was conducted among young adults using a custom-designed online questionnaire. The research sample consisted of 729 participants. The data were analyzed using ANOVA, and due to the nature of the data, Wilcoxon tests were also employed to examine differences. The most frequently purchased fruits among young adults were bananas and apples; however, strawberries and raspberries were the most favored. The most preferred apple cultivars were bicolored (e.g., Jonagored) and red (e.g., Gala Royal), while yellow cultivars (e.g., Golden Delicious) were perceived as the least attractive. Young consumers favored apples that were juicy, firm, and moderately sweet. This study demonstrated that the skin color of an apple was associated with expectations regarding its firmness and sweetness. Apples with intense coloration (dark red and green) received the highest valuations, particularly when organically grown. In contrast, apples from conventional production systems were valued below their market price by young consumers, indicating the need for strategies aimed at enhancing their perceived value.

## 1. Introduction

Apples are not a homogeneous product; there is a vast number of cultivars that differ in appearance (e.g., skin or flesh color) and sensory attributes. The industry aims to offer consumers cultivars that meet their expectations while simultaneously considering technological aspects such as storability, shelf life, and resistance to diseases and physical damage. Consequently, the apple industry continuously seeks new cultivars to increase demand and profitability. Therefore, it is essential for the industry to gain a comprehensive understanding of the traits that consumers value most.

Objective quality refers to the physical characteristics of a product and is measured by engineers and food technologists using instruments and physical and chemical techniques [1]. Among the objective attributes of apples considered in consumer preference studies are cultivar, size, and quality grade [2]. Appearance plays a crucial role in the immediate purchasing decision. Not only color or shape but even minor blemishes or damages on the fruit significantly influence the purchase of both organic apples [3,4] and conventional apples [4].

Subjective quality is the assessment of sensory properties by consumers [1]. It has been shown that quality (taste, aroma, and texture) is one of the most important factors influencing the decision to purchase apples [5]. Quality was even more important than price [6]. In the literature, one can come across choice experiments involving the sensory evaluation of apples [7,8,9,10]. According to previous research findings, internal sensory attributes (firmness, sweetness) influence a higher valuation of apples, while the results of studies on the external appearance of apples do not provide conclusive results. Consumers are willing to pay more for higher crispness [2,7,11], sweetness [2], as well as for subjectively perceived flavor [12,13,14], which is a combination of sweetness and acidity [13]. In some studies, an impact of socio-demographic characteristics has been observed, but it is weaker than that of sensory attributes [15]. There are, however, examples of studies demonstrating the significance of socio-demographic characteristics. It has been shown that 88% of consumers of Asian ethnicity identified themselves as sweet apple consumers, whereas only 55% of consumers of European ethnicity reported the same [16]. In the case of the Estonian sample, it was demonstrated that interest in the sensory attributes of apples was higher among women and individuals under the age of 25 [17]. It was also shown that apple consumption habits vary significantly with age, with higher consumption observed in older age groups [18]. Studies have shown that consumers decide to purchase apples based on experiences related to internal attributes, such as taste [19]. It has also been demonstrated that purchase preferences developed on the basis of sensory characteristics are stable and assigned greater importance [20].

Consumer demand can motivate apple producers to choose safer and more environmentally friendly apple cultivation methods and thus sell at higher prices [21]. Building a strong and positive brand image can increase demand for healthy products [15]. According to research findings, consumers are willing to pay more for food labeled as organic, “green,” or environmentally friendly and produced in sustainable systems [22,23]. The COVID-19 pandemic has also influenced consumer attitudes, making them aware of the importance of purchasing environmentally friendly products [24]. With regard to the apple market, it has been proven that consumers were willing to pay more for apples that had traceable production information, lower pesticide usage, and were cultivated using organic fertilizers [25]. Advertising shapes ecological attitudes and consumer awareness and directly influences the purchasing behavior of eco-friendly products [26]. Pro-environmental behavior is more influenced by education than by reaching maturity; therefore, the more consumers are exposed to information on this topic, the more pronounced their engagement becomes [27].

The consumption of fruits is an important part of a healthy diet and also brings health benefits. The trend towards a healthy lifestyle significantly increases the demand for healthy food [28,29]. This does not lead to an increase in apple demand in all countries.

Consumption of apples is decreasing in many European countries [30] due to the belief that apples have poorer sensory qualities and dissatisfaction among some consumers with popular apple cultivars and changes in income, prices, and demographic factors [31]. The decline in apple consumption is also attributed to greater competition from other types of fruits compared to the past, as well as the belief that some apples are not the healthiest choice [30]. According to the authors, the decline in apple consumption in Poland results from the increasing wealth of societies. Consumers in these countries more frequently choose more expensive fruits, including citrus fruits, bananas, berries, and others (e.g., exotic fruits) from a wide and growing assortment [32,33].

The declining consumption of apples in Poland, along with the increase in production, is causing difficulties in managing apple harvests. Therefore, it seems important to understand the fruit market preferences, especially for apples, of young people who are entering adulthood and managing their own budget, whether received or earned. During their studies, some individuals move out of their family homes to cities where they study, thus being forced to make purchasing decisions as in their own households. This period is when purchasing habits are formed, which may continue later in life. Understanding the preferences of young people is necessary to shape the supply to match the demand or to be able to influence demand through well-thought-out marketing campaigns. The aim of this study was to investigate the preferences of young adults in the Polish apple market in response to the declining consumption of these fruits.

Based on previous research findings and market observations in Poland, the authors formulated the following research questions:

1. Which fruits are most liked by young adults, and which are most frequently purchased?
2. How are apples positioned in terms of likability, purchase frequency, and trendiness compared to other fruits?
3. What color of apples is considered the most delicious?
4. What are young adults’ preferences regarding the sweetness, firmness, and juiciness of apples?
5. Do preferences regarding the color of apples influence the expected levels of sweetness, firmness, and juiciness of the fruit?
6. Does the appearance of apples—particularly their color—influence the perceived value of the fruit?
7. Do young consumers assign a higher value to organically grown apples compared to conventionally grown ones?
8. Is the consumer-perceived valuation of apples higher than their current market prices?

## 2. Materials and Methods

This study involved 729 respondents recruited from first- and second-year university students. Data collection took place from 16 October to 20 December 2024. A structured online questionnaire was created for this study and distributed among students from various universities in Poland using the CAWI (Computer-Assisted Web Interview) technique. At the beginning of the questionnaire, participants were presented with an informed consent form for participation in this study and a consent form for the processing of personal data, along with a statement ensuring their understanding of how the data would be used for research purposes. Only individuals who accepted the study conditions were allowed to participate. Participants were also informed about the anonymity of their responses and their right to withdraw at any stage of this study.

According to data from the Ministry of Science and Higher Education, 684.3 thousand students were enrolled in Poland in the first and second years of first-cycle and long-cycle studies [34]. The sample size required to obtain representative results with a 5% margin of error and a 95% confidence level should be 385 people.

This study utilized a questionnaire with closed-ended questions, including both single-choice and multiple-choice options, as well as open-ended questions. The questionnaire included three questions that featured images of apple cultivars differing, among other characteristics, in skin color (green—Granny Smith; yellow—Golden Delicious; bicolored—Ligol, Jonagored; red—Gala Royal; dark red—Red Delicious). Subjective taste preferences were assessed by the participants using a 5-point Likert scale in terms of sweetness (1—sour; 5—sweet), texture (1—soft; 5—hard/crispy), and juiciness (1—not juicy; 5—very juicy).

Respondents were between 17 and 24 years of age (M = 19.94; SD = 1.46), of whom 56.5% were women, 43.2% were men, and 0.3% represented other genders. Respondents were primarily recruited from larger cities (over 500,000 inhabitants), accounting for 43.3%, and to a lesser extent from rural areas (23.5%). The remaining respondents originated from smaller cities, particularly those with populations ranging from 11,000 to 50,000 inhabitants (14.8%).

Data regarding the amount of disposable budget for personal expenses (from all sources, including donations) were provided by 80% of respondents, while 20% refused to disclose the amount. Monthly disposable income for personal expenses ranged from PLN 0 to PLN 10,000, with a mean (M) of PLN 2511 (approximately USD 612.22) and a standard deviation (SD) of PLN 2003.72 (approximately USD 488.52). A larger percentage of respondents (98.8%) specified the share of self-earned funds in their budgets. On average, self-earned funds constituted 56.3% of students’ budgets (M = 70%, SD = 39%).

A one-way ANOVA was conducted to analyze apple taste preferences depending on the preferred color of apples. The assumption of normality distribution was assessed using the Shapiro–Wilk test at a significance level of α = 0.05. Depending on whether the assumption of homogeneity of variances was met (Levene’s test), appropriate post hoc tests (Games–Howell; LSD) were applied at a significance level of α = 0.05. Taste preferences were examined using both verbal and pictorial questions, and the obtained results were compared.

Respondents were asked to evaluate six apple cultivars based on the provided images, with price options ranging from PLN 3.50/kg to PLN 7.50/kg (in increments of PLN 0.50/kg). The same images used in the evaluation of the tastiest apple were presented but in a different order. The images were displayed simultaneously, allowing respondents to compare the entire set of apples. Respondents were given the same task in the next question, using the same images, but this time, the apples were described as coming from organic cultivation.

To compare apple price valuations based on skin color, production system (conventional vs. organic), and average retail prices observed in stores, the Wilcoxon signed-rank test for paired samples was utilized. The selection of this non-parametric test was justified by the failure to meet the assumptions required for parametric analyses [35,36]. The Wilcoxon signed-rank test for dependent samples enables the determination of whether one category systematically yields higher or lower values compared to another [37]. Data analysis and hypothesis testing in this study were carried out using SPSS version 25.

## 3. Results

Among the respondents, 93.6% consumed apples, while the remaining respondents (6.7%) did not eat these fruits.

The fruits most frequently indicated as favorites by respondents consuming fruit were strawberries (16.0%) and raspberries (14.4%), both of which are seasonal fruits cultivated in Poland for centuries. Apples were indicated by 12.2% of respondents, while blueberries and bananas were chosen slightly more frequently. Altogether, these five fruits were preferred by 67.5% of respondents, and adding the next two fruits, watermelon and citrus, this cumulative percentage increased to 86.4%.

For 43.1% of respondents, the most frequently purchased fruits were bananas, while apples were most frequently bought by 26% of respondents and citrus fruits by 14.2%. Among the fruits indicated as most frequently purchased were also berries, such as blueberries (5.0%), raspberries (4.3%), and strawberries (3.7%). Only 3.8% of respondents most frequently bought other fruits than those mentioned.

Strawberries, blueberries, bananas, apples, raspberries, and citrus fruits were identified by respondents as the most fashionable fruits, together accounting for over 84% of all answers to the question on fashionable fruit consumption. Strawberries ranked highest, with 20.8% of participants naming them the most fashionable, followed by blueberries (17.9%), bananas (13.3%), apples (13.0%), raspberries (11.3%), and citrus fruits (8.1%).

In response to the question regarding the preferred apple color among respondents, most indicated a preference for bicolored apples (32.0%), followed closely by red apples (31.2%). A smaller percentage of respondents preferred green apples (21.3%). Dark red apples were less popular (11.6%), while yellow apples were the least popular (4.0%). Based on the results of the questions with pictures of six types of apples, 37.2% of respondents identified bicolored apples as the tastiest. The highest number of respondents selected the bicolored Jonagored apple (28.4%) as the most delicious-looking. The next most appealing were the red apples (25.5%) and green apples (21.8%). Dark red apples were selected by 12% of respondents, followed by bicolored Ligol apples at 8.8%. Yellow apples were considered the least delicious-looking, chosen by only 3.4% of respondents.

The mean rating for preferred apple firmness on a scale from 1 (soft) to 5 (hard) was 3.93 (SD = 1.015). The expected acidity level of apples in the studied sample was 3.22 (SD = 0.997) on a scale from 1 (sour) to 5 (sweet), while the mean rating for juiciness was 4.16 (SD = 0.857) on a scale from 1 (low juiciness) to 5 (very juicy).

The relationship between apple color preferences and expectations regarding the taste of the fruit was examined. Table 1 presents the results of taste preferences according to the preferred apple skin color as indicated in the verbal question.

Due to the lack of homogeneity of variance in the analyzed variables (Levene’s test was statistically significant), Welch’s and Brown–Forsythe’s tests were applied, which proved to be statistically significant for acidity (F (4; 143.662) = 32.386; *p* < 0.001; ω^2^ = 0.19) and softness (F (4; 144.640) = 5.538; *p* < 0.001; ω^2^ = 0.31). Considering the obtained omega-squared (ω^2^) value, the observed effect can be regarded as strong.

The conducted post hoc comparison using the Games–Howell test revealed significant differences in the expected softness of apples between individuals who prefer green-skinned apples (M = 4.19; SD = 1.086) and those who prefer apples with yellow (M = 3.33; SD = 1.144; *p* = 0.008) and red skin colors (M = 3.79; SD = 0.964; *p* = 0.005). Additionally, statistically significant differences (*p* = 0.04) were found in the expected softness of apples between individuals who prefer bicolored apples (M = 4.02; SD = 0.926) and those who prefer yellow apples (Table 2).

The conducted post hoc comparison using the Games–Howell test revealed significant differences (*p* < 0.001) in the expected acidity of apples between individuals who prefer green-skinned apples (M = 2.41; SD = 1.05) and those who prefer apples of other skin colors: yellow (M = 3.67; SD = 1.04), bicolored (M = 3.26; SD = 0.79), red (M = 3.54; SD = 0.84), and dark red (M = 3.59; SD = 0.98). Additionally, statistically significant differences (*p* = 0.005) were found in the expected acidity of apples between individuals who prefer bicolored apples and those who prefer red apples (Table 3).

No statistically significant differences were found in terms of juiciness, which can be interpreted as consumers having similar expectations regarding this variable regardless of the external appearance of the apple—namely, they expect apples to be highly juicy (on average, 4.07). Only in the case of yellow apples were the expectations lower; however, the sample size was small, and the standard deviation was too large to demonstrate statistical significance (Table 1).

In the analysis of the relationship between softness, acidity, and juiciness depending on the selection of the most appetizing apple in the pictorial question (Table 4), statistically significant differences were found for softness and acidity, similar to the results obtained in the verbal question.

In the case of softness, Levene’s test for homogeneity of variance was not statistically significant (F (5; 676) = 3.75; *p* = 0.002, η^2^ = 0.27). Eta-squared (η^2^) suggests a strong influence of the examined factor on the dependent variable.

The conducted post hoc comparison using the LSD test revealed significant differences in the expected softness of apples between individuals who prefer green-skinned apples (M = 4.22; SD = 1.032) and those who prefer yellow (M = 3.70; SD = 1.032; *p* = 0.020), bicolored Ligol (M = 3.70; SD = 1.139; *p* < 0.001), bicolored Jonagored (M = 3.92; SD = 0.933; *p* = 0.007), red (M = 3.87; SD = 0.959; *p* = 0.002), and dark red (M = 3.80; SD = 1.082; *p* = 0.03) apples.

Due to the lack of homogeneity of variance in the variable acidity (Levene’s test was statistically significant), Welch’s and Brown–Forsythe’s tests were applied, which proved to be statistically significant for acidity F (5; 151.160) = 21.576; *p* < 0.001; ω^2^ = 0.157. Considering the obtained omega-squared (ω^2^) value, the observed effect can be regarded as strong.

The conducted post hoc comparison using the Games–Howell test revealed significant differences in the expected acidity of apples between individuals who prefer green-skinned apples (M = 2.48; SD = 1.037) and those who prefer apples of other skin colors: yellow (M = 3.43; SD = 1.161; *p* = 0.011), bicolored Ligol (M = 3.22; SD = 0.804; *p* < 0.001), bicolored Jonagored (M = 3.35; SD = 0.809; *p* < 0.001), red (M = 3.53; SD = 0.891), and dark red (M = 3.55; SD = 0.971; *p* < 0.001).

The highest prices (PLN 4.92/kg) among conventionally produced apples, according to respondents, were achieved by dark red apples (Red Delicious) (Table 5). The median and mode prices for these apples reached PLN 5.00/kg. In contrast, the lowest valuation (PLN 4.02/kg) was given to the bicolored apples (Ligol), whose mode price was just PLN 3.50/kg, with a median of PLN 4.00/kg. Yellow apples were rated low in terms of price. Their perceived value exceeded only that of the bicolored Ligol apples. It was examined whether there were statistically significant differences in the valuation of apples of different colors (cultivars). The results of the Wilcoxon test indicated that for each compared pair, differences in valuation (prices) were statistically significant (*p* < 0.001) for conventionally produced apples (Table 6).

For organic apples, statistically significant differences in valuation were observed in most cases (*p* < 0.001), as determined by the non-parametric Wilcoxon test. The only exception was the comparison between bicolored Jonagored apples and red apples, where no statistically significant difference was detected (Table 7). The patterns observed for organic apples were consistent with those identified for conventionally produced apples (Table 5). Dark red apples (Red Delicious) received the highest average valuation (PLN 5.27/kg), although both the median and mode remained at levels comparable to those recorded for conventionally grown counterparts. In contrast, the bicolored Ligol variety received the lowest valuation (PLN 4.56/kg) despite its median and mode being higher than those of its conventionally produced equivalent.

The results of the Wilcoxon signed-rank test conducted to compare the price valuations of apples with the same skin color (cultivars) from conventional and organic production revealed statistically significant differences for all six cultivars (*p* < 0.001). The test statistics indicated strong effects in all cases (Table 8). The greatest differences in the valuation of apples of the same variety (color), depending on the production system, were observed for bicolored Ligol apples (PLN 0.54/kg, approx. USD 0.132/kg) and yellow apples (PLN 0.50/kg, approx. USD 0.122/kg). The smallest difference was noted for dark red apples (PLN 0.35/kg, approx. USD 0.085/kg).

In the question concerning the perceived health benefits of apples from organic farming, respondents were asked to assess whether they believe organic apples are healthier than those produced through conventional methods. The largest share of respondents, 40.5%, considered organic apples to be healthier, while 20.7% expressed the opposite opinion. A significant portion (38.9%) declared that they had no opinion on the matter.

The pricing of conventional apples was compared with the current retail prices of dessert apples (Table 9). In the literature, prices were available for the studied apple colors (cultivars) in the conventional version [38]. The range of organic fruits in Poland is more limited, and reliable data were not available for all examined types of apples.

The results obtained using the Wilcoxon signed-rank test indicated that only in the case of dark red apples did respondents’ valuation not differ significantly from retail prices valid during the study period. The valuation of bicolored Jonagored apples was close to current retail prices, yet according to the applied statistical test, the difference was statistically significant (*p* = 0.031). For the remaining apple colors, the Wilcoxon signed-rank test showed significant differences at a significance level of *p* < 0.001. The valuation of all apples was significantly lower than current market prices.

In another question, respondents were also asked to evaluate the current retail price of class I-quality dessert apples, which was PLN 4.50 per kilogram (approximately USD 1.09/kg) and PLN 1.15 per piece (approximately USD 0.28/piece). The majority of respondents (43%) considered this price appropriate, while over a quarter (26.5%) rated it as high, 9.1% as low, and 21.4% had no opinion on the matter. In a separate question, respondents were asked whether they would prefer to purchase higher-quality (premium) apples at a price of PLN 6.00/kg (PLN 1.50/piece; approx. USD 1.52/kg, USD 0.38/piece) or class I apples at a lower price of PLN 4.50/kg (PLN 1.25/piece; approx. USD 1.14/kg, USD 0.32/piece). The results indicate that 35.6% of respondents expressed a preference for premium apples, while an identical proportion chose class I apples. The remaining 28.7% had no opinion on the matter. In the question regarding which fruit is the most valuable and should be priced the highest—among the given options of bananas, oranges, and apples—the majority of respondents (61%) indicated oranges, whereas apples were considered the most valuable by only 8.6% of participants.

## 4. Discussion

Among respondents who reported not consuming apples, a significant proportion indicated having a food allergy to this fruit. Among fruits, the apple (*Malus domestica*) is the second most common food allergen after peach, with a prevalence of 4.2% [39,40]. According to research findings, in Southern European countries (e.g., Spain, Italy), the percentage of consumers sensitized to apples is significantly higher, reaching up to 15%, whereas in Northern European countries, this prevalence is lower and does not exceed 5% [41,42]. This percentage is significant from the producers’ perspective, as it limits market potential. It cannot be unequivocally determined whether all respondents who did not consume apples were allergic or avoided fruits for other reasons. Nevertheless, the authors found the obtained result unexpectedly high, given that nearly 7% of respondents reported not consuming fruits.

The data suggest that the most preferred fruits are either seasonal fruits available from domestic production only at certain times of the year (strawberries, raspberries, blueberries) or those available all year round (bananas, apples). Seasonal fruits such as strawberries and raspberries are also available to a limited extent and at higher prices outside the harvest season in Poland. In the case of blueberries, their availability lasts throughout the year, but outside the Polish harvest season, their prices are significantly higher. The year-round supply of blueberries is the result of the development of production and export of these fruits worldwide over recent decades [43,44,45]. In Poland, they are imported in spring from Southern Europe and in autumn and winter from the Southern Hemisphere (Chile, Peru) [46].

Bananas, apples, and citrus fruits were indicated as favorite fruits by a smaller combined percentage of respondents (23.6%), yet these fruits were reported as the most frequently purchased by as many as 83.3% of the surveyed consumers. For apples, the number of respondents identifying them as the most frequently purchased was more than twice as high as the number of those who declared apples as their favorite fruit. An even greater disparity was observed for bananas, with nearly 3.5 times more respondents indicating bananas as the most frequently purchased fruit compared to those who considered bananas their favorite fruit.

Based on the responses regarding favorite and most frequently purchased fruits, it can be concluded that consumers tend to choose fruits that are widely available throughout the year. Bananas and apples are present in retail chains all year round. Apples, which are extensively cultivated in Poland, are the least expensive among these fruits. Bananas, on the other hand, are several dozen percent more expensive. Therefore, the higher popularity of bananas is likely driven by factors other than price alone. Potential explanations include their consistent taste profile and predictable quality, which are conditions less consistently met in the case of apples [47]. In the case of citrus fruits, the supply peaks mainly during the winter season when imports originate from Southern Europe, coinciding with the period of their highest sales. In contrast, imports from the Southern Hemisphere generate lower consumer interest and are associated with higher prices. Blueberries, compared to the most frequently purchased fruits, are the most expensive—even during the domestic harvest season in Poland. Outside the season, the prices of imported blueberries, raspberries, and strawberries rank among the highest of all fruits available on the market. Price may, therefore, be one of the main factors limiting the frequency of their purchase, especially considering that a significantly higher proportion of respondents indicated these fruits as their favorites. In the case of strawberries and raspberries, lower purchase frequency may also be attributed to their limited availability, as they are not present in the market year-round but mainly during the domestic harvest season and early spring when they are imported from Southern Europe.

When comparing these results with respondents’ favorite fruits, strawberries stood out as both the most fashionable and the most liked, underlining their strong appeal among Polish consumers. Blueberries also ranked high in both categories and were perceived as more fashionable than liked, possibly due to their association with health trends and “superfood” status [48,49,50,51,52,53,54,55,56,57,58,59]. Strawberries are also frequently classified as a superfood in global studies [60,61,62,63,64,65,66]; however, in the Polish context, this designation is more commonly applied to blueberries. In contrast, raspberries were more often chosen as a favorite than for fashionability. This may be due to the fact that the health benefits of raspberries are less well-known, and they are less frequently classified as superfoods, which is discussed in the literature, particularly in older publications [67,68]. For bananas, apples, and citrus fruits, preferences and perceived fashionability were relatively aligned. The results obtained regarding favorite and trendy fruits can be used in marketing.

Among young adults, bicolored apples were the most popular, followed by red and green cultivars. Notably, green apples received a relatively high number of indications as both the favorite and the most delicious (21–22%), especially considering that their availability on the Polish market is significantly lower compared to bicolored apples. The proportion of yellow apples selected by respondents was notably low (3–4%) in both the verbal and image-based survey items. In the visual component, the yellow apple was represented by the Golden Delicious cultivar. This limited consumer preference aligns with the declining market performance of Golden Delicious in the fresh fruit segment, a trend that has been increasingly evident over recent seasons [63,64]. In the present study, dark red apples (Red Delicious) were selected more frequently only in comparison to yellow apples (Golden Delicious). This suggests the potentially limited marketability of such fruit, which aligns with global trends, as the production of Red Delicious apples has been declining for years in major production regions (e.g., the United States) [69]. Production of the Gala variety has increased on all continents, making it the most popular apple variety in international trade [33]. The qualities of this variety translate into higher consumer valuations compared to other apple cultivars [11]. However, in the present study, Gala (a red apple) was not rated higher by consumers, presumably due to its similarity in appearance to some bicolored apples commonly found on the Polish market. Habit and familiarity play a significant role in consumer choices in the apple market [6].

In the case of green, yellow, and dark red apples, the number of participants who indicated a preference for these fruits was relatively consistent across both the verbal and pictorial question formats. However, the group that selected bicolored apples as the most appetizing in the image-based question was 16.5% larger compared to the verbal question. This discrepancy is likely attributable to differences in perception between visually presented apples and participants’ mental representations or prototypes of apples. The greater prevalence of bicolored apples in retail markets in Poland may result in their images aligning more closely with the commonly imagined prototype of an apple. Indeed, the majority of apples sold in Poland are bicolored, with the degree of red pigmentation varying according to the specific cultivar and growing conditions [70,71,72,73,74]. Moreover, the image-based question offered two types of bicolored apples, whereas the verbal question presented only one option, which may have further contributed to the higher selection rate in the pictorial format. Conversely, the number of respondents who identified red apples as the most appetizing was 22% lower in the photo-based question compared to the verbal format. The red apple depicted was of the Gala Royal variety, which is less frequently available in Polish retail markets, as it is primarily produced for export [75]. Additionally, the blurred coloration (striped streaks) and the atypical shape (more flattened) of this variety may have negatively influenced participants’ perceptions in the image-based assessment.

The Polish young adults surveyed indicated that apples should ideally possess a high level of juiciness, a firm texture, and a balanced, moderate sweetness. Taste is a combination of sweetness and acidity and is an important factor determining consumer acceptance [10]. When formulating conclusions from this study, it is important to recognize that consumer evaluations are inherently subjective. As noted in previous research, sweetness in consumer assessments is a subjective attribute that is not directly linked to sugar content [10]. Prior studies have shown that consumers tend to place higher value (in terms of willingness to pay) on sweeter and juicier fruits, whereas experimental studies have not yielded conclusive results regarding firmness [4]. In other research, consumers were willing to pay more for apples with higher firmness [11] and sweetness in the case of Gala and Red Delicious cultivars [2,69]. In the present study, sweetness did not emerge as a particularly desirable attribute, in contrast to juiciness and firmness. This may be due to differing apple preferences across countries or regions of the world. The topic merits further, in-depth investigation using standardized research methods to better understand consumer preferences in various parts of the world. Experimental studies, in particular, may be especially useful, as they could provide valuable insights for apple suppliers.

Preferences regarding apple skin color in the verbal question were associated with expectations concerning fruit firmness. Young adults who prefer green-skinned apples expect them to be firmer than those who favor yellow or red cultivars. Similarly, individuals who prefer bicolored apples anticipate greater fruit firmness compared to those who prefer yellow apples. People who find green-skinned apples the most appetizing tend to expect firmer fruit compared to those who prefer apples with any other skin color in the pictural question. When comparing the results of preference questions regarding fruit softness between verbal and pictorial assessments, statistically significant differences were found in the two combinations. Individuals who find green apples the most appetizing expect less soft fruit compared to those who consider red or yellow apples the most appetizing. In this case, the pictorial question revealed differences in evaluation between the imagined apple and the one presented in the image. The photograph of the green apple resulted in statistically significant differences compared to all other colors, whereas the verbal question—relying on imagination—produced significant differences only in the case of red and yellow, which are associated with lower perceived firmness, and between bicolored and yellow.

When it comes to acidity, individuals who consider green apples the most delicious expected the fruit to be more sour than consumers who preferred all other fruits, both in the pictorial and verbal questions. Therefore, the preferred green color of the apple skin is associated with the expectation of a more sour taste of the fruit. A significant difference was also observed in the word question between those who favor bicolored apples and those who prefer red apples in terms of expected acidity. This can be explained by the fact that in the verbal question, participants imagined a bicolored apple, whereas, in the pictorial question, they were presented with two bicolored options, one of which contained a significant amount of red— a color associated with a sweeter expected taste. The second option was less frequent, which may have contributed to the lack of statistically significant differences.

High juiciness was the most desired apple characteristic among respondents, as indicated by the highest mean rating and the lowest standard deviation, reflecting the greatest consistency in preferences. Respondents also clearly favored firm apples. A lower declared preference was observed in relation to apple sweetness, as participants, on average, expected apples to be sweet and sour. However, consumer expectations in this category were the most divergent, as evidenced by the highest standard deviation.

According to the research findings, skin color influences the perceived value of apples. Fruits with uniform, intense coloration (red or green) were rated higher, whereas those that were blurred red coloration, bicolored, and yellow were rated lower. The lowest valuations were assigned to bicolored apples, particularly those with a greater proportion of green. The differences in the valuation of individual apple cultivars, both from conventional and organic production, were statistically significant. The sole exception was the comparison between bicolored Jonagored apples and red apples, for which the difference in valuation was not statistically significant in the case of organic. The lack of statistically significant differences in the valuation of red apples and bicolored Jonagored apples in the organic version was due to their similar valuation in the conventional version (a difference of only PLN 0.10/kg), combined with generally higher valuations of organic apples and a greater increase in prices for the cheaper fruits.

These findings confirm that organic labeling significantly increases the perceived value of apples across all analyzed cultivars, although the strength of this effect varies depending on the apple type. This suggests that apple cultivars perceived as less valuable may benefit the most from being offered as organically produced. In line with previous research, advertising influences purchasing behavior in the market for eco-friendly products [26], while eco-labels do not have a direct impact but significantly affect the ecological attitudes and awareness of millennials purchasing eco-friendly products [26,76,77]. Ecological issues (green knowledge, attitude, environmental knowledge, and purchase intention for green products) can be used as a brand marketing strategy to increase purchase intention among consumers [28]. The main concerns of (Chinese) consumers are quality certificates and information regarding synthetic fertilizers and pesticides [21]. Subjective norms influence green purchase intentions. Although millennials in college have high purchase intentions, the overwhelming majority have not resulted in actual purchases, as consumption habits are the main factor influencing purchases [29].

The results regarding the perceived health benefits of apples from organic farming indicate a slight predominance of the belief that organic apples are healthier compared to those who remain undecided on this issue. This may reflect a limited level of consumer knowledge concerning the differences between organic and conventional farming practices or skepticism towards the current organic labeling present on the market. At the same time, the percentage of respondents who did not perceive organic apples as healthier was approximately half that of those who believed they were healthier. This perception was reflected in the valuation of organic apples, which was significantly higher than that of conventionally grown fruit. Interestingly, no statistically significant difference was found in the valuation of organic apples depending on whether respondents perceived organically produced apples as healthier.

The results confirm that a significant proportion of respondents perceive apples as fruits of lower value than their market price would suggest. One possible explanation is the quality of apples available on the market, which is often assessed as unsatisfactory [78]. Another contributing factor may be the perception of apples as commonplace fruits, which may lead to a diminished perceived value. This study demonstrated that, compared to oranges and bananas, apples are perceived as less valuable. This may be due to the fact that consumers perceive apples as less healthy compared to other fruits [30].

When evaluating apple prices, it is also important to note that in the 2024/25 season, retail apple prices were 20–30% higher [79], which may have influenced price assessments. On the other hand, the price evaluation scale, ranging from PLN 3.50 to 7.50/kg, was designed in such a way that average market prices were positioned closer to the lower end of the scale. This may have led to inflated price assessments among consumers lacking knowledge of actual apple prices, consistent with the central tendency bias [80,81]. Dark red apples were evaluated by respondents at a level similar to prevailing retail prices. All other apple types (cultivars) were valued below market prices. Consumer price sensitivity regarding apples may vary depending on income level. In the present study, no statistically significant relationship between income and apple valuation was identified; however, the population of young adults studied is specific, as not all participants were independently managing their own households. A portion of the respondents derived income solely from personal sources. The relationship between apple prices—which are among the lowest-priced fruits—and the frequency of apple consumption appears to be a promising avenue for future research. This is particularly relevant given that previous studies conducted on a sample of residents in Chinese metropolitan areas demonstrated that more than half (53.5%) of consumers were price-insensitive, while just under a quarter (24.8%) exhibited price sensitivity [25].

The fact that over one-third of respondents (35.6%) declared a willingness to pay a higher price for apples with superior quality parameters—i.e., premium class—should be noted by market participants. This may indicate the existence of a market niche that could be capitalized on by apple suppliers. Empirical evidence indicates that quality surpasses price in importance [6]. However, this is not certain, as previous studies have demonstrated that quality does not always translate into purchase intention due to various barriers [15]. On the other hand, according to other research findings, regardless of socio-economic characteristics, consumers are willing to pay a higher price for apples that are tastier, more visually appealing, and more aromatic, as well as for traditional cultivars [8]. In the present study, however, Polish young adults valued apples below the market price of class I quality despite the fact that the apples depicted in the photographs were of high (premium) quality and appeared significantly more appealing than those typically available on the market.

Analyzing the results of the conducted study gives rise to new research questions. Are the obtained results comparable among older generations? Is the perception of sweetness consistent among respondents? Would offering sweeter apples—i.e., those with lower acid content and higher sugar levels—be met with consumer acceptance and lead to increased willingness to purchase?

## 5. Conclusions

This study revealed a significant discrepancy between consumer preferences and actual purchasing behavior. While bananas and apples turned out to be the most frequently purchased fruits, strawberries and raspberries were identified as the most liked. Apples are neither the most preferred nor the most “trendy” fruit. Their market position is likely due to their wide availability and relatively low price.

The most preferred apples among young adults were bicolored cultivars, which also constitute the largest share of supply in Poland, as well as red cultivars. A relatively high proportion of respondents indicated a preference for green apple cultivars, especially when compared to their availability in the retail market, which is a noteworthy insight for market stakeholders. Dark red cultivars were less popular among young adults. Yellow apples proved to be the least attractive.

According to young adults, apples should be highly juicy, firm, and moderately sweet. It can be inferred that the greener the apple, the more sourness consumers tend to expect. Conversely, red and yellow skin is generally associated with higher sweetness. The apple’s skin color is also linked to expectations about its texture. Consumers expect green apples to be firmer than red and yellow ones, which are perceived as softer. Regardless of color preferences, young adults tend to favor juicy apples.

The color of the apple’s skin affects its perceived value among young consumers. Apples with intense coloration (dark red and green) were assigned higher values. The lowest valuations were given to bicolored apples, especially those with a greater proportion of green.

Apples are evaluated differently depending on the cultivar and the production system. Organic apples receive higher valuations. Within the same production system, cultivars with intense coloration (red and green) are rated more highly by young adults.

Based on the study results regarding apple prices, it can be concluded that on the one hand, consumers perceive apples as overpriced, while on the other, some express a willingness to pay more for higher quality.

A particularly important finding is that young consumers value apples below their actual market price. To counteract the declining consumption of apples, efforts should be made to enhance their perceived value. One possible approach is to offer higher-quality fruits that meet consumer preferences. Another direction could be the promotion of organically grown apples.

The results obtained in this study may be useful for market participants in selecting appropriate apple cultivars for cultivation, determining the type of production, and addressing technological factors that affect the quality of fruits offered on the retail market.

## Figures and Tables

**Table 1:** Descriptive statistics of variance analysis of taste preferences (acidity, softness, juiciness) depending on the preferred apple color (word question).

Category	N	Mean	Standard Deviation	Standard Error	95% CI Lower Bond	95% CI Upper Bond	Min.	Max.
Softness								
Green	145	4.19	1.086	0.090	4.01	4.36	1	5
Yellow	27	3.33	1.144	0.220	2.88	3.79	1	5
Bicolored	218	4.02	0.926	0.063	3.89	4.14	1	5
Red	213	3.79	0.964	0.066	3.66	3.92	1	5
Dark red	79	3.82	1.071	0.121	3.58	4.06	1	5
Overall	682	3.93	0.986	0.038	3.86	4.00	1	5
Acidity								
Green	145	2.41	1.045	0.087	2.24	2.59	1	5
Yellow	27	3.67	1.038	0.200	3.26	4.08	1	5
Bicolored	218	3.28	0.878	0.059	3.16	3.40	1	5
Red	213	3.54	0.838	0.057	3.42	3.67	1	5
Dark red	79	3.59	0.981	0.110	3.38	3.81	1	5
Overall	682	3.22	0.997	0.038	3.15	3.30	1	5
Juiciness								
Green	145	4.17	1.041	0.087	3.99	4.35	1	5
Yellow	27	3.41	1.127	0.217	2.96	3.87	1	5
Bicolored	218	4.00	0.902	0.061	3.88	4.13	1	5
Red	213	4.19	0.856	0.059	4.07	4.31	1	5
Dark red	79	4.19	0.976	0.110	3.97	4.42	1	5
Overall	682	4.07	0.933	0.033	4.00	4.13	1	5

Source: Own elaboration.

**Table 2:** Multiple comparisons of softness (dependent variable) of Games–Howell post hoc test.

Dependent Variable		Mean Difference	Std. Error	Sig.	95% Confidence Interval	
					Lower Bound	Upper Bound
Green	Yellow	**0.853** *	0.238	0.008	0.17	1.54
	Bicolored	0.168	0.110	0.545	−0.13	0.47
	Red	**0.393** *	0.112	0.005	0.09	0.70
	Dark red	0.363	0.151	0.117	−0.05	0.78
Yellow	Green	**−0.853** *	0.238	0.008	−1.54	−0.17
	Bicolored	**−0.685** *	0.229	0.040	−1.35	−0.02
	Red	−0.460	0.230	0.289	−1.13	0.21
	Dark red	−0.489	0.251	0.307	−1.20	0.23
Bicolored	Green	−0.168	0.110	0.545	−0.47	0.13
	Yellow	**0.685** *	0.229	0.040	0.02	1.35
	Red	0.225	0.091	0.099	−0.02	0.47
	Dark red	0.196	0.136	0.604	−0.18	0.57
Red	Green	**−0.393** *	0.112	0.005	−0.70	−0.09
	Yellow	0.460	0.230	0.289	−0.21	1.13
	Bicolored	−0.225	0.091	0.099	−0.47	0.02
	Dark red	−0.029	0.137	1.000	−0.41	0.35
Dark Red	Green	−0.363	0.151	0.117	−0.78	0.05
	Yellow	0.489	0.251	0.307	−0.23	1.20
	Bicolored	−0.196	0.136	0.604	−0.57	0.18
	Red	0.029	0.137	1.000	−0.35	0.41

* The result is statistically significant. Source: Own elaboration.

**Table 3:** Multiple comparisons of acidity (dependent variable) of Games–Howell post hoc test (word question).

Dependent Variable		Mean Difference	Std. Error	Sig.	95% Confidence Interval	
					Lower Bound	Upper Bound
Green	Yellow	**−1.253** *	0.218	0.000	−1.88	−0.63
	Bicolored	**−0.848** *	0.102	0.000	−1.13	−0.57
	Red	**−1.121** *	0.104	0.000	−1.41	−0.84
	Dark red	**−1.181** *	0.140	0.000	−1.57	−0.79
Yellow	Green	**1.253** *	0.218	0.000	0.63	1.88
	Bicolored	0.405	0.207	0.310	−0.19	1.01
	Red	0.131	0.208	0.969	−0.47	0.73
	Dark red	0.072	0.228	0.998	−0.58	0.72
Bicolored	Green	**0.848** *	0.102	0.000	0.57	1.13
	Yellow	−0.405	0.207	0.310	−1.01	0.19
	Red	**−0.274** *	0.079	0.005	−0.49	−0.06
	Dark red	−0.333	0.123	0.057	−0.67	0.01
Red	Green	**1.121** *	0.104	0.000	0.84	1.41
	Yellow	−0.131	0.208	0.969	−0.73	0.47
	Bicolored	**0.274** *	0.079	0.005	0.06	0.49
	Dark red	−0.060	0.124	0.989	−0.40	0.28
Dark Red	Green	1.181 *	0.140	0.000	0.79	1.57
	Yellow	−0.072	0.228	0.998	−0.72	0.58
	Bicolored	0.333	0.123	0.057	−0.01	0.67
	Red	0.060	0.124	0.989	−0.28	0.40

* The result is statistically significant. Source: Own elaboration.

**Table 4:** Descriptive statistics of variance analysis of taste preferences (acidity, softness, juiciness) depending on the preferred apple color (picture question).

Category	N	Mean	Standard Deviation	Standard Error	95% CI Lower Bond	95% CI Upper Bond	Min.	Max.
Softness								
Green	149	4.22	1.032	0.085	4.05	4.39	1	5
Yellow	23	3.70	1.105	0.230	3.22	4.17	2	5
Bicolored Ligol	60	3.70	1.139	0.147	3.41	3.99	1	5
Bicolored Jonagored	194	3.92	0.933	0.067	3.79	4.05	1	5
Red	174	3.87	0.959	0.073	3.73	4.02	2	5
Dark Red	82	3.80	1.082	0.120	3.57	4.04	1	5
Overall	682	3.93	1.015	0.039	3.86	4.01	1	5
Acidity								
Green	149	2.48	1.037	0.085	2.32	2.65	1	5
Yellow	23	3.43	1.161	0.242	2.93	3.94	1	5
Bicolored Ligol	60	3.22	0.804	0.104	3.01	3.42	2	5
Bicolored Jonagored	194	3.35	0.809	0.058	3.24	3.47	1	5
Red	174	3.53	0.891	0.068	3.40	3.66	1	5
Dark Red	82	3.55	0.971	0.107	3.34	3.76	1	5
Overall	682	3.22	0.997	0.038	3.15	3.30	1	5
Juiciness								
Green	149	4.17	0.860	0.070	4.04	4.31	1	5
Yellow	23	3.70	1.329	0.277	3.12	4.27	1	5
Bicolored Ligol	60	4.05	0.982	0.127	3.80	4.30	1	5
Bicolored Jonagored	194	4.15	0.844	0.061	4.04	4.27	1	5
Red	174	4.27	0.673	0.051	4.17	4.37	2	5
Dark Red	82	4.16	0.936	0.103	3.95	4.36	1	5
Overall	682	4.16	0.857	0.033	4.10	4.23	1	5

Source: Own elaboration.

**Table 5:** Measures of central tendency for apple pricing, depending on color and production system.

Conventional Apples		Bicolored Ligol	Dark Red	Yellow	Green	Red	Bicolored Jonagored
N	Valid	682	682	682	682	682	682
	Missing	47	47	47	47	47	47
Mean		4.02	4.92	4.25	4.61	4.49	4.39
Median		4.00	5.00	4.00	4.50	4.50	4.50
Mode		3.50	5.00	4.00	4.50	4.00	4.00
**Organic Apples**		**Bicolored Ligol**	**Dark Red**	**Yellow**	**Green**	**Red**	**Bicolored Jonagored**
N	Valid	682	682	682	682	682	681
	Missing	47	47	47	47	47	48
Mean		4.56	5.27	4.75	5.04	4.91	4.87
Median		4.50	5.00	4.50	5.00	5.00	4.50
Mode		4.50	5.00	4.50	5.00	4.50	4.50

Source: Own elaboration.

**Table 6:** Comparison of apple valuation from conventional production using the Wilcoxon signed-rank test for dependent samples.

Comparison of Pairs of Apples	1 with 2	1 with 3	1 with 4	1 with 5	1 with 6
Total N	682	682	682	682	682
Test Statistic	158,315.5	75,146.0	123,633.5	104,325.5	74,268.0
Standard Error	4033.359	2817.690	3509.313	3122.437	2461.080
Standardized Test Statistic	18.148	7.361	14.954	13.754	11.958
Asymptotic Sig. (2-sided Test)	*p* < 0.001	*p* < 0.001	*p* < 0.001	*p* < 0.001	*p* < 0.001
**Comparison of Pairs of Apples**	**2 with 3**	**2 with 4**	**2 with 5**	**2 with 6**	**3 with 4**
Total N	682	682	682	682	682
Test Statistic	21,944.5	38,929.5	33,280.5	34,139.5	10,6071.5
Standard Error	3828.867	3258.692	3479.332	3686.100	3377.255
Standardized Test Statistic	−15.075	−7.813	−10.656	−11.217	11.121
Asymptotic Sig. (2-sided Test)	*p* < 0.001	*p* < 0.001	*p* < 0.001	*p* < 0.001	*p* < 0.001
**Comparison of Pairs of Apples**	**3 with 5**	**3 with 6**	**4 with 5**	**4 with 6**	**5 with 6**
Total N	682	682	682	682	682
Test Statistic	85,097.5	74,143.5	52,035.5	48,329.5	37,324.5
Standard Error	3187.904	3089.532	3203.181	3402.345	2521.404
Standardized Test Statistic	6.892	4.451	−3.699	−5.932	−4.002
Asymptotic Sig. (2-sided Test)	*p* < 0.001	*p* < 0.001	*p* < 0.001	*p* < 0.001	*p* < 0.001

Source: Own elaboration.

**Table 7:** Comparison of apple valuation from organic production using the Wilcoxon signed-rank test for dependent samples.

Comparison of Pairs of Apples	1 with 2	1 with 3	1 with 4	1 with 5	1 with 6
Total N	682	682	682	682	681
Test Statistic	130,058.5	63,163.0	97,518.0	76,319.5	59,459.5
Standard Error	3551.233	2480.001	3012.372	2553.715	2133.615
Standardized Test Statistic	16.285	6.960	13.052	11.489	10.545
Asymptotic Sig. (2-sided Test)	*p* < 0.001	*p* < 0.001	*p* < 0.001	*p* < 0.001	*p* < 0.001
**Comparison of Pairs of Apples**	**2 with 3**	**2 with 4**	**2 with 5**	**2 with 6**	**3 with 4**
Total N	682	682	682	681	682
Test Statistic	21,220.5	34,626	28,823	32,637	81,583
Standard Error	3275.295	2782.324	2974.096	3187.030	2843.778
Standardized Test Statistic	−13.413	−6.444	−9.555	−9.331	9.475
Asymptotic Sig. (2-sided Test)	*p* < 0.001	*p* < 0.001	*p* < 0.001	*p* < 0.001	*p* < 0.001
**Comparison of Pairs of Apples**	**3 with 5**	**3 with 6**	**4 with 5**	**4 with 6**	**5 with 6**
Total N	682	681	682	681	681
Test Statistic	67,009.5	58,766	44,597.5	42,098	30,667.5
Standard Error	2710.385	2602.891	2887.688	2903.525	1950.347
Standardized Test Statistic	5.837	4.025	−3.966	−4.643	−1.777
Asymptotic Sig. (2-sided Test)	*p* < 0.001	*p* < 0.001	*p* < 0.001	*p* < 0.001	*p* = 0.076

Source: Own elaboration.

**Table 8:** Comparison of the pricing of apples of the same skin color (cultivars) from conventional and organic production using the Wilcoxon signed-rank test for dependent samples.

	Bicolored Ligol	Dark Red	Yellow	Green	Red	Bicolored Jonagored
Total N	682	682	682	682	682	681
Test Statistic	107,447	88,025.5	98,751	98,805	99,899	102,573
Standard Error	3059.282	2917.672	2899.57	3079.697	3111.29	3054.745
Standardized Test Statistic	15.621	10.878	14.889	12.473	12.46	14.049
Asymptotic Sig. (2-sided Test)	*p* < 0.001	*p* < 0.001	*p* < 0.001	*p* < 0.001	*p* < 0.001	*p* < 0.001

Source: Own elaboration.

**Table 9:** Comparison of the pricing of apples of different colors (cultivars) from conventional production with the average retail prices in effect at the end of 2024, using the one-sample Wilcoxon signed-rank test.

	Bicolored Ligol	Dark Red	Yellow	Green	Red	Bicolored Jonagored
Total N	682	682	682	682	682	682
Average Retail Price	4,50	4,99	4,69	4,99	4,99	4,50
Test Statistic	36,514	114,137	47,148	68,688	47,433	105,450
Standard Error	5083.725	5129.407	5107.578	5120.854	5110.706	5112.860
Standardized Test Statistic	−15.724	−0.451	−13.569	−9.327	−13.505	−2.152
Asymptotic Sig. (2-sided Test)	*p* < 0.001	0.652	*p* < 0.001	*p* < 0.001	*p* < 0.001	0.031

Source: Own elaboration.

## Data Availability

The original contributions presented in the study are included in the article, further inquiries can be directed to the corresponding author.
